# Hormone metabolism pathway genes and mammographic density change after quitting estrogen and progestin combined hormone therapy in the California Teachers Study

**DOI:** 10.1186/s13058-014-0477-8

**Published:** 2014-12-11

**Authors:** Eunjung Lee, Jianning Luo, Yu-Chen Su, Juan Pablo Lewinger, Fredrick R Schumacher, David Van Den Berg, Anna H Wu, Leslie Bernstein, Giske Ursin

**Affiliations:** Department of Preventive Medicine, Keck School of Medicine, University of Southern California/Norris Comprehensive Cancer Center, 1441 Eastlake Avenue, Los Angeles, CA 90089 USA; Department of Population Sciences, Beckman Research Institute, City of Hope, 1500 East Duarte Road, Duarte, CA 91010 USA; Department of Nutrition, University of Oslo, PB 1046 Blindern, 0317 Oslo, Norway; Cancer Registry of Norway, PB 5313 Majorstuen, 0304 Oslo, Norway

## Abstract

**Introduction:**

Mammographic density (MD) is a strong biomarker of breast cancer risk. MD increases after women start estrogen plus progestin therapy (EPT) and decreases after women quit EPT. A large interindividual variation in EPT-associated MD change has been observed, but few studies have investigated genetic predictors of the EPT-associated MD change. Here, we evaluate the association between polymorphisms in hormone metabolism pathway genes and MD changes when women quit EPT.

**Methods:**

We collected mammograms before and after women quit EPT and genotyped 405 tagging single nucleotide polymorphisms (SNPs) in 30 hormone metabolism pathway genes in 284 non-Hispanic white participants of the California Teachers Study (CTS). Participants were ages 49 to 71 years at time of mammography taken after quitting EPT. We assessed percent MD using a computer-assisted method. MD change was calculated by subtracting MD of an ‘off-EPT’ mammogram from MD of an ‘on-EPT’ (that is baseline) mammogram. Linear regression analysis was used to investigate the SNP-MD change association, adjusting for the baseline ‘on-EPT’ MD, age and BMI at time of baseline mammogram, and time interval and BMI change between the two mammograms. An overall pathway and gene-level summary was obtained using the adaptive rank truncated product (ARTP) test. We calculated ‘*P* values adjusted for correlated tests (*P*_ACT_)’ to account for multiple testing within a gene.

**Results:**

The strongest associations were observed for rs7489119 in *SLCO1B1*, and rs5933863 in *ARSC*. SLCO1B1 and ARSC are involved in excretion and activation of estrogen metabolites of EPT, respectively. MD change after quitting was 4.2% smaller per minor allele of rs7489119 (*P* = 0.0008; *P*_ACT_ = 0.018) and 1.9% larger per minor allele of rs5933863 (*P* = 0.013; *P*_ACT_ = 0.025). These individual SNP associations did not reach statistical significance when we further used Bonferroni correction to consider the number of tested genes. The pathway level summary ARTP *P* value was not statistically significant.

**Conclusions:**

Data from this longitudinal study of EPT quitters suggest that genetic variation in two hormone metabolism pathway genes, *SLCO1B1* and *ARSC*, may be associated with change in MD after women stop using EPT. Larger longitudinal studies are needed to confirm our findings.

**Electronic supplementary material:**

The online version of this article (doi:10.1186/s13058-014-0477-8) contains supplementary material, which is available to authorized users.

## Introduction

Mammographic density (MD) is a measure of the amount of epithelium and stroma relative to the amount of fat tissue in the breast. MD is one of the strongest biomarkers of breast cancer risk [1] and is associated with a number of breast cancer risk factors [2]. Combined estrogen plus progestin therapy (EPT) is an established risk factor of breast cancer [3-9]. Data from a randomized clinical trial, the Women’s Health Initiative (WHI), confirmed that an EPT regimen consisting of conjugated equine estrogens (CEE) and medroxyprogesterone acetate (MPA) [9] increased breast cancer risk. EPT use has also been associated with a substantial increase in MD, by 3 to 5% in the Postmenopausal Estrogen/Progestin Interventions (PEPI) trial, depending on the regimen [10,11], and by approximately 7% in the WHI trial [12]. In both studies, a large interindividual variation was observed [10-12]. In the PEPI trial, approximately 20% of women in the EPT group experienced a one-step increase in BI-RADS grade, which represents 14 to 18% increase in density [10]. This enormous change was not observed in others in the EPT group, but some increase was observed in a larger number of women [10,11]. Importantly, the increase in MD was positively correlated with post-treatment increases in serum levels of estrone (E1) [13], estrone sulfate (E1S) [14], and progestogen levels [15]. These findings suggest that genetic factors influencing absorption and metabolism of EPT may be important in predicting EPT-associated MD change.

Similarly, MD decreases after discontinuing hormone therapy [16,17]. In a randomized trial of short-term cessation of hormone therapy, MD significantly decreased in the EPT cessation group, but with an interindividual variation in MD decrease [17,18]. In the EPT cessation group, MD decreased by ≥7.5% in 24% of women; decreased by 3% to <7.5% in another 24%; changed little (<3%) in 30%; and increased by >3% in 23% [18]. None of the measured lifestyle factors modified the magnitude of the MD decrease, and the authors concluded that genetic factors could be important determinants. Considering that increases in MD are associated with higher breast cancer risk [19] and that decreases in MD are associated with reduced breast cancer risk [19,20], identifying genetic determinants of MD change associated with EPT use and quitting is important to understand breast cancer risk in current or former EPT users.

While there have been efforts to identify genetic determinants of MD in large cross-sectional samples [21,22], few studies have investigated the genetic predictors of longitudinal MD changes associated with EPT use. In a small sample from a clinical trial of EPT use where four single nucleotide polymorphisms (SNPs) in four hormone pathway genes were investigated, two SNPs (Val432Leu in *CYP1B1* and L311V in *AKR1C4*) were associated with MD change after starting EPT [23]. A longitudinal study based on the European Prospective Investigation into Cancer and Nutrition (EPIC) cohort suggested that genetic variation in progesterone receptor gene (*PGR*) modified the effect of hormone therapy on MD change [24]. Data from the PEPI trial did not support this association: none of the 23 tested tagging SNPs in the *PGR* were associated with MD change in EPT treatment arms [15]. Each of these studies investigated less than five genes [15,23,24], and only the PEPI study genotyped more than two SNPs in each gene [15]. In the current study, we investigated the association between 405 SNPs in 30 hormone metabolism pathway genes and MD change after stopping EPT use using data from the California Teachers Study (CTS) mammographic density subcohort.

## Materials and methods

### California Teachers Study (CTS)

The CTS is a prospective cohort of 133,479 current and former female public school teachers and other public school professionals, who were members of the California State Teachers Retirement System in 1995. At baseline, cohort participants completed a mailed questionnaire providing detailed information on reproductive history, oral contraceptive use, hormone therapy use and personal medical history including any previous diagnosis of cancer. A detailed description of the CTS is available [25]. The CTS was approved by the Institutional Review Board at each collaborating institution: the Cancer Prevention Institute of California, the University of California, Irvine, the University of Southern California, and the City of Hope in accord with assurances filed with and approved by the US Department of Health and Human Services.

### Mammographic density substudy

The CTS mammographic density substudy was conducted to evaluate the role of genetic polymorphisms on MD changes occurring after women initiate or stop using EPT. We selected 1,420 women from among those who responded to a follow-up questionnaire collected around 2000 to 2001 and who were aged 40 to 60 years at cohort enrollment, lived in California and had not had a cancer diagnosis since enrolling in the study, newly initiated EPT use during the interval between enrollment and completion of the third questionnaire collected around 2000 to 2001, had a mammogram in the past two years, and were not participating in another CTS substudy. These initial selection criteria were used to recruit women who were most likely to have had recent mammograms before and after they started EPT use. Characteristics of the 1,420 women who were selected are described in Additional file 1. Since it was assumed that many in the CTS stopped using EPT following the publication of WHI results in June 2002 [26], the eligibility criteria described above were also expected to identify women who had mammograms before and after they quit EPT. We sent out letters to 1,420 CTS participants to solicit participation in this mammogram substudy and to inform women we would be contacting them by telephone. We spoke with 1,272 women (89.6%), and identified 21 women who were ineligible for this study (seven with a previous cancer diagnosis and 14 who did not meet the mammography screening criterion). Of the 1,251 eligible women, 247 women did not participate in this mammographic density substudy. Reasons for non-participation were refusals (n = 111), not providing written informed consent (n = 134), withdrawal of consent (n = 1), and not completing telephone interview (n = 1); a total of 1,004 women were included in the study. We conducted a telephone interview with eligible participants to obtain updated information on menstrual history and hormone use. We collected mammograms for 993 of the 1,004 women, and excluded 29 because we could not determine whether they were using hormone therapy at the time of mammogram. On average, eight mammograms for each participant were available to us. Some mammography facilities enclosed information on menstrual history and hormone use of each woman at the time of each mammogram. This information was available for approximately 50% of all participants and for approximately 40% of their mammograms. When we compared this information with the interview-based hormone therapy information, approximately 40% of these women had some inconsistency in the years of hormone therapy use between the two data sources. About 67% of the inconsistencies was due to ±1 year difference in the year when they started or stopped using hormone therapy; several years of difference were noted in other women. We considered the information collected from medical records at the time of mammography to be more accurate than women’s recall at the time of study enrollment (telephone interview), and therefore used the information from the mammography facilities when available. The mammographic density substudy of the CTS was approved by the University of Southern California institutional review board, and all participants provided written informed consent.

### Selection of mammograms

To determine MD changes following the changes in women’s EPT use, we intended to select one ‘on-EPT mammogram’ taken while the participant was using EPT and one ‘off-EPT mammogram’ taken while the participant was not using any type of hormone therapy, either EPT or ET. Many EPT users in the CTS quit EPT use after the publication of WHI results in June 2002 [26]. We also realize that many women cannot recall exactly when they stopped hormone therapy. We therefore decided to select the on-EPT mammogram that was taken before July 1 2002, to minimize misclassification of EPT use status. When selecting off-EPT mammograms, we preferred mammograms that were taken after 2003. The implication was that we would mostly measure MD changes after ‘stopping’ EPT use rather than changes after ‘starting’ EPT use. Second, it has been shown that EPT-related MD changes occur predominantly within the first 12 months after starting EPT use and remain constant for at least the next two years [10]. Therefore, we preferred to select off-EPT mammograms taken as close as possible to the year of on-EPT mammograms but with at least a one-year interval between the on-EPT and off-EPT mammograms. After applying these criteria, we selected on-EPT mammograms of 578 women and off-EPT mammograms of 757 women. Both an on-EPT mammogram and an off-EPT mammogram were available for 422 women, comprising the longitudinal set of this mammogram substudy.

### Construction of EPT quitters’ dataset

Of the 422 in the longitudinal set, 371 women were quitters, and 51 women were starters. Many women start using EPT during their menopausal transition, when ovarian estrogen levels are decreasing but are not as low as in postmenopausal women [27,28]. It is possible that off-EPT mammograms of some EPT starters were taken while they were still undergoing the menopausal transition, although women reported that they were postmenopausal at that time. In such cases, the measurement of MD change before and after starting EPT use (that is increasing hormone levels) would be complicated by the MD change associated with the menopausal transition (that is decreasing hormone levels). Upon inspection of the menopausal status data for the starters, we could not exclude the possibility that the off-EPT mammogram of the majority of the starters were taken before they became completely postmenopausal. If this was the case, the data from these starters are likely to measure a decline in MD when going from a pre- or perimenopausal off-EPT state to a postmenopausal state on EPT, rather than to measure an increase in MD when going from a postmenopausal off-EPT state to a postmenopausal on-EPT state. Therefore, we decided to restrict the current study to the 371 EPT quitters in the longitudinal set.

### Mammographic density assessment

The methods for MD measurements used for this study have been described previously [11,13]. We digitized the images using a Cobrascan CX812T scanner (Radiographic Digital Imaging, Torrance, CA, USA) at a resolution of 150 pixels/inch (59 dots/cm). MD was assessed by one of the authors (GU) on scanned images using the USC Madena method, a validated computer-assisted, quantitative technique [10]. The total area of the breast was assessed by a research assistant trained by GU. MD, or the percent mammographic density, was calculated as the absolute density divided by the total area of the breast (as a percent). Readers were blinded to EPT use and which mammograms belonged to the same patient. We estimated change in MD by subtracting MD of off-EPT mammogram from that of on-EPT mammogram. For quality assurance, we included random blinded duplicates of 183 mammograms. Correlation between duplicate MD measures was excellent (R = 0.96).

### Specimen collection and DNA extraction

We mailed an Oragene DNA self-collection kit (DNA Genotek, Kanata, ON, Canada) to the participant and return postage-paid mailing materials. Of the 371 women eligible for the current study, 328 women provided sufficient amount of samples for DNA extraction. We extracted DNA using the Oragene protocol (DNA Genotek).

### Tagging SNP selection and genotyping

We used the same tagging SNPs selected for a CTS-nested case-control study of breast cancer [29]. Briefly, we selected linkage disequilibrium (LD) tagging SNPs across each gene, 20 kb upstream of 5′ untranslated region (UTR) and 10 kb downstream of 3′ UTR, using the Snagger software [30] and the TagSNPs program [31,32]. We aimed to capture all common SNPs (minor allele frequency (MAF) of at least 5%) in whites with minimum pairwise r^2^ of at least 0.80. For some genes, due to space limitation of our genotyping platform, we included a few selected SNPs instead of complete lists of tagging SNPs.

Genotyping of the selected 455 SNPs were performed using the Illumina Golden Gate Assay (Illumina, Inc., San Diego, CA, USA) in the University of Southern California Core Facility. We excluded 38 SNPs with a call rate <90%, five SNPs with Hardy-Weinberg equilibrium *P* value <0.001, and seven SNPs with MAF < 1%, leaving 405 SNPs for analyses. About 6% of the 328 genotyped samples (n = 19) had a genotyping success rate (call rate) of less than 90% and were excluded from the analyses. The majority of the remaining 309 samples were non-Hispanic whites (n = 284). Due to the small sample size of women in other ethnic groups, we restricted the analysis to 284 non-Hispanic whites. The genotyping concordance rate was >99.9% based on 15 duplicate samples with a call rate >90%.

### Statistical analysis

We used linear regression to examine the association between genotype and change in MD (that is ‘on-EPT density’ minus ‘off-EPT density’), adjusting for age and body mass index (BMI) (kg/m^2^) at on-EPT mammogram, time interval and BMI change between the two mammograms, and ‘on-EPT density’. These adjustment variables were chosen because age and BMI are strong determinants of MD [1], and baseline MD could be related to the absolute level of MD decrease [18]. An additive genetic model was used, which estimate the difference in the outcome variable (that is change in MD) per copy of the minor allele of each SNP (that is modeled as 0, 1, or 2 copies of the minor allele). The individual SNP *P* values were corrected for multiple testing within each gene using the *P*_ACT_ method [33], which takes into account correlation due to LD. In order to capture gene and pathway level effects that may not be detectable through any single SNP, we also performed gene-based and pathway-based tests using the adaptive rank truncated product (ARTP) method [34]. ARTP adaptively combines single SNP *P* values within a gene region or a pathway to obtain a single test statistic for the gene or pathway and assesses significance of the test by a permutation procedure. Unlike a multiple testing procedure like *P*_ACT_, which accounts for multiple SNP tests in order to properly control the type I error, ARTP combines information across SNPs within a gene or a pathway in order to increase the power to detect a gene or pathway level effect.

### Imputation

For a gene region that contains the most significant finding, we imputed genotype data for all SNPs reported in the 1000 Genomes project [35]. As the reference panel, we used the haplotypes of 1,092 samples (all populations) from the release version 3 of the 1000 Genomes Project Phase I [35]. Combining reference data from all populations helps improve imputation accuracy of low-frequency variants [36]. The haplotypes were phased using SHAPEIT2 [37]. We imputed genotypes in the selected gene region to the 1000 Genomes reference panel using IMPUTE2 [38]. We used the ‘certainty’ and ‘info’ metrics as the imputation quality measure, with cutpoints of >0.9 and >0.5 for each metric, respectively. We also excluded imputed SNPs with MAF < 0.01. We used the genotype probabilities in a dosage format as opposed to the best genotype calls in the association tests.

Parity induces substantial proliferation, differentiation, and subsequent involution of breast tissue cells [39]. It is known that nulliparous women have higher MD than parous women [40,41], and that the association between MD and breast cancer risk may be stronger in nulliparous women than in parous women [42]. Therefore, we conducted exploratory analyses stratified by parity. Statistical tests for interaction were evaluated by introducing product terms with genotype and conducting Wald tests. We also conducted a subgroup analysis restricted to women who were aged 56 or older at time of on-EPT mammogram. This analysis helps to exclude the possibility that the genotype-MD change association is mainly driven by the MD changes related to menopausal transition rather than EPT-related MD changes. In a large population-based US study, >97% of those with natural menopause were postmenopausal by age 56 years [7]. Longitudinal data from the Minnesota Breast Cancer Family Study also suggest that non-hormone users show large decline in MD until her early 50s, and the age-related decline slows down from her late 50s [43]. We also performed subgroup analyses and statistical tests of interaction by baseline BMI (<25 kg/m^2^, ≥25 kg/ m^2^) and BMI change (<±1 kg/m^2^, greater than 1 kg/m^2^ increase or decrease in BMI) between the two mammograms.

## Results

Women who were included in the final analysis (284 non-Hispanic white EPT quitters) were similar to those interviewed but not included in the analysis with respect to several factors except menopausal status (Table 1). Those included in the analysis were more likely to be peri- or postmenopausal at cohort enrollment. The time interval between the two mammograms was ≤3 years for about 50% of the 284 EPT quitters, and >3 to 5 years for another 37% (Table 1). Mean (± standard deviation (SD)) of the MD change between the two mammograms was 4.0% (±7.0%). The absolute amount of MD decrease was positively associated with baseline age (regression coefficient = 0.20, *P* = 0.04), baseline BMI (regression coefficient = 0.19, *P* = 0.016), baseline MD (regression coefficient = 0.17, *P* < 0.0001), longer time interval (regression coefficient = 0.43, *P* = 0.078), and larger BMI increase (regression coefficient = 0.46, *P* = 0.056) between the two mammograms, when all of these variables were included in the regression model.

**Table 1:** **Comparison of characteristics of women participating in the California Teachers Study mammographic density substudy who were included in the analyses with those of participants who were excluded from the analyses**

**Characteristics**	**Interviewed participants not included in the current analysis (n = 720)**	**Interviewed participants included in the current analysis (non-Hispanic white EPT quitters) (n = 284)**	***P*** **values**
**At cohort enrollment**			
Age (mean ± SD)	49.9 ± 4.2 (range 40-60)	50.5 ± 3.8 (range 41-60)	0.07
BMI (kg/m^2^)	24.6 ± 4.8 (range 17-50)	24.7 ± 4.9 (range 17-47)	0.68
White (N (%))	624 (87%)	284 (100%)	
Nulliparous women (N (%))	141 (20%)	63 (22%)	0.33
Menopausal status			
Premenopausal	432 (60%)	155 (55%)	0.012
Perimenopausal	87 (12%)	55 (19%)	
Postmenopausal	201 (28%)	74 (26%)	
Ever had breast biopsy (N (%))	108 (15%)	40 (14%)	0.71
Positive 1st degree family history of breast cancer (N (%))	64 (9%)	37 (13%)	0.053
**At mammogram substudy enrollment**			
Age at interview (mean ± SD)	62 ± 4.0 (range 52-71)	62 ± 3.6 (range 53-72)	0.11
BMI (kg/m^2^)	26.2 ± 5.2 (range 17-49)	25.7 ± 5.0 (range 18-47)	0.16
Positive 1st degree family history of breast cancer (N (%))	112 (16%)	51 (18%)	0.43
Number of mammograms in the past 10 years	8.8 ± 2.3 (range 2-20)	8.9 ± 2.2 (range 2-18)	0.59
**At time of mammography evaluated for density**			
Age at time of mammogram while taking EPT		56 ± 4 (range 45-67)	
Age at time of mammogram while off EPT		60 ± 5 (range 49-71)	
BMI at time of mammogram while taking EPT		25.7 ± 5.2 (range 18-47)	
BMI at time of mammogram while off EPT		25.8 ± 5.0 (range 18-48)	
Years on EPT at time of mammogram while taking EPT			
<1 year		15 (5%)	
1- < 4 years		121 (43%)	
4- < 7 years		112 (39%)	
≥7 years		36 (13%)	
Time interval between two mammograms (years)			
≤3 years		141 (50%)	
4-5 years		106 (37%)	
6-9 years		37 (13%)	

EPT, estrogen and progestin combined therapy; BMI, body mass index.

The overall ARTP test for the entire set of 405 SNPs in the hormone metabolism pathway genes was not statistically significant (*P* = 0.49; data not shown). When we applied this method to each gene separately, the gene-level ARTP test *P* values were 0.02 and 0.04, respectively, for *ARSC* and *SLCO1B1* (Table 2), suggesting that genetic variation in *ARSC* and *SLCO1B1* may be associated with EPT-associated MD change. However, when we considered the number of genes tested and applied a Bonferroni correction, these gene-level ARTP test *P* values were not statistically significant.

**Table 2:** **Gene-level summary** ***P*** **values associated with mammographic density change after quitting EPT use**

**Gene**	**Number of SNPs genotyped**	**Gene-level association** ***P*** **value** *
*AKR1C4*	1	0.44
*AR*	6	0.39
*ARSC*	2	0.02
*COMT*	21	0.14
*CYP11A*	12	0.64
*CYP19A1*	2	0.80
*CYP1A1/CYP1A2*	5	0.97
*CYP1B1*	15	0.61
*CYP21A2*	2	0.13
*CYP2C9*	16	0.73
*CYP3A4*	3	0.46
*ESR1*	13	0.81
*ESR2*	22	0.68
*HSD17B1*	5	0.11
*HSD17B2*	24	>0.99
*HSD17B4*	24	0.49
*HSD17B5/AKR1C3*	38	0.85
*HSD3B1*	4	0.14
*HSD3B2*	10	0.97
*PGR*	32	0.81
*SHBG*	13	0.60
*SLCO1B1 (SLC21A6)*	38	0.04
*SRD5A1*	24	0.80
*SULT1A1/SULT1A2*	6	0.26
*SULT1E1*	18	0.50
*UGT1A8*	43	0.99
*UGT2B17*	1	0.63
*UGT2B7*	5	0.52

*Based on the adaptive rank truncated product (ARTP) statistics. EPT, estrogen and progestin combined therapy; SNP, single nucleotide polymorphisms.

When we tested the association between MD and each individual SNP, only two SNPs, rs7489119 in *SLCO1B1* and rs5933863 in *ARSC*, showed statistically significant associations after correcting for multiple testing within each gene (Table 3). The MD decrease after quitting EPT was 4.2% smaller per minor allele (A allele) of rs7489119 (in *SLCO1B1*). The least squares mean of MD change for CC genotype carriers was 4.5% (that is 4.5% decrease in MD after quitting EPT), adjusted for the covariates described in the Materials and Methods section. In contrast, the least squares mean for the CA or AA genotype carriers was −0.2% (that is 0.2% increase in MD after quitting EPT; data not shown). When we performed association tests for 575 imputed SNPs in the *SLCO1B1* region, only rs79640916 and rs78854974 were associated with MD change with *P* values of 0.0006 and 0.0007 (not corrected for multiple testing), respectively. Rs79640916 and rs78854974 are located in the intron regions of *SLCO1B1*, 23 kb and 40 kb away from rs7489119, and in LD with rs7489119 (r^2^ = 0.65 for both SNPs) in European populations based on 1000 Genomes data [35]. None of the other imputed SNPs were associated with MD change with a *P* value <0.001 (Additional file 2).

**Table 3:** **SNPs that are statistically significantly associated with EPT-associated mammographic density change after correcting for multiple testing** ^*^

**SNP (major/minor allele)**	**Gene**	**Minor allele frequency**	**N (WW/WV/VV)**	**beta**	**SE**	***P***	***P***_**ACT**_^¶^
rs7489119 (C/A)	*SLCO1B1*	0.041	258/23/1	−4.22	1.25	0.0008	0.018
rs5933863 (G/A)	*ARSC*	0.15	200/79/5	1.87	0.75	0.013	0.025

*Based on linear regression model adjusting for age and BMI (kg/m^2^) at time of on-EPT mammogram, time interval and BMI change between the two mammograms, and mammographic density of on-EPT mammogram. Additive genetic model was used. ^¶^Multiple testing corrected *P* value; *P*_ACT_ (*P* values adjusted for correlated tests) within each gene was calculated using the methods by Conneely and Boehnke [33]. EPT, estrogen and progestin combined therapy; SNP, single nucleotide polymorphisms; SE, standard error; BMI, body mass index.

For rs5933863 (*ARSC*), the MD decrease after quitting EPT was 1.9% larger per minor allele (A allele) (Table 3). The least squares means for GG carriers was 3.4% while it was 5.7% for GA or AA carriers (data not shown). In other words, GG carriers had 3.4% decrease in MD after quitting EPT, and GA or AA carriers had 5.7% decrease in MD after quitting EPT. However, if we further applied Bonferroni corrections considering the number of genes tested, none of these associations remained statistically significant. The results for all investigated SNPs are presented in Additional file 3.

In our exploratory subset analysis restricted to 64 nulliparous women, rs2077647 in *ESR1* and rs9605030 in *COMT* were associated with EPT-related density changes, which remained statistically significant after correcting for multiple testing at the gene level (both *P*_ACT_ < 0.05; Table 4). The minor allele of rs2077647 was associated with 3.9% larger density decrease after quitting EPT. The minor allele of rs9605030 was associated with 4.5% larger density decrease after quitting EPT. *P* values for interaction with parity for these two SNPs were 0.003 and 0.011, respectively (uncorrected for multiple testing).

**Table 4:** **SNPs that are statistically significantly associated with EPT-associated mammographic density change in either nulliparous (n = 63) or parous (n = 219) women after correcting for multiple testing at gene level** ^**¶**^

**SNP**		**Minor**	**Nulliparous**	**(n = 63)**			**Parous**	**(n = 219)**			
**(major/minor allele)**	**Gene**	**allele frequency**	**N (WW/WV/VV)**	**Beta (SE)**	***P***	***P***_ACT_^*^	**N (WW/WV/VV)**	**Beta (SE)**	***P***	***P***_ACT_^*^	***P*** **for interaction** ^**†**^
rs2077647 (T/C)	*ESR1*	0.49	18/30/15	3.92 (1.06)	0.0005	0.006	56/118/43	−0.40 (0.63)	0.53	>0.99	0.003
rs9605030 (C/T)	*COMT*	0.14	40/21/2	4.47 (1.42)	0.0025	0.043	166/48/4	0.69 (0.88)	0.44	>0.99	0.011
rs5933863 (G/A)	*ARSC*	0.15	46/17/0	1.26 (1.88)	0.51	0.75	152/62/5	2.00 (0.81)	0.014	0.027	0.82

^¶^Based on linear regression model adjusting for age and BMI (kg/m^2^) at time of on-EPT mammogram, time interval and BMI change between the two mammograms, and mammographic density of on-EPT mammogram. Additive genetic model was used. *Multiple testing corrected *P* value; *P*_ACT_ (*P* values adjusted for correlated tests) within each gene was calculated using the methods by Conneely and Boehnke [33]. ^†^ *P* values for interaction were not corrected for multiple testing. SNP, single nucleotide polymorphisms; EPT, estrogen and progestin combined therapy; SE, standard error; BMI, body mass index.

Among 219 parous women, only rs5933863 in *ARSC* had a gene-level *P*_ACT_ <0.05, but this SNP showed similar magnitude of association with MD change in nulliparous women (*P* for interaction = 0.82; Table 4).

When the analysis was restricted to women who were aged 56 or older at the time of their on-EPT mammogram (n = 143), the association with rs7489119 (*SLCO1B1*) became stronger: the minor allele of rs7489119 was associated with 6.2% smaller density decrease after quitting EPT (*P* = 0.0003; *P*_ACT_ = 0.007; data not shown). The association with rs5933863 (*ARSC*) was similar in magnitude, although this association was not statistically significant (*P*_ACT_ >0.05; data not shown).

EPT-related MD changes occur predominantly within the first 12 months after starting EPT use and remain rather constant for at least the next two years [10]. When we excluded 15 women whose on-EPT mammogram was taken within one year from starting EPT, the results were essentially identical to those we have presented. Further adjustment for duration of EPT at time of on-EPT mammogram did not change the results. We did not observe any evidence of effect modification by BMI at baseline or BMI change between the two mammograms.

## Discussion

In this longitudinal study of EPT quitters, overall genetic variation in the 30 hormone metabolism pathway genes was not associated with density change. However, at the gene-level, we found some evidence that two hormone metabolism pathway genes, namely *SLCO1B1* and *ARSC*, were associated with MD change after women quit EPT use. The few SNPs previously proposed to be associated with EPT-associated density change, including SNPs in *PGR* (rs10895068) [24], *AKR1C4* (rs17134592) [23], *CYP1B1* (rs1056836) [23], were not associated with MD change in this study. Rs10895068 and rs17134592 were genotyped in this study, and rs1056836 was tagged with three SNPs in LD (r^2^ = 0.74-0.76). To our knowledge, this study is the first to systematically investigate hormone metabolism pathway genes as genetic determinants of longitudinally assessed MD change after women quit EPT use.

Data from WHI and PEPI clinical trials have shown that EPT use for one year increased MD by 3 to 7% [10-12]. In a randomized trial, two-month suspension of EPT decreased MD to a larger extent (by 2.8%) than was observed in the comparison group who continued EPT use [17]. However, in all studies, large interindividual variation in the MD change was noted after introducing [10-12] or stopping EPT use [17]. Increases and decreases in MD have been associated with higher and lower, respectively, risk of breast cancer in a study of women who were not using hormone therapy [19]. In addition, in a nested case-control study within a breast cancer prevention trial, decrease in MD was a good predictor of tamoxifen-induced reduction in breast cancer risk [20]. Further, genetic variation in a tamoxifen metabolizing enzyme *CYP2D6* has been associated with MD change following tamoxifen treatment [44]. Thus it seems reasonable that understanding the genetic determinants of the interindividual variability in MD changes in response to EPT use or quitting can help predict breast cancer risk in EPT users or former users.

Our finding that one SNP (rs7489119) in *SLCO1B1* may be involved in determining EPT-related MD change is novel and biologically plausible. *SLCO1B1*, also known as *SLC21A6*, is a solute carrier organic anion transporter gene expressed in the liver. SLCO1B1 transports a variety of endogenous and exogenous substrates from the blood into the hepatocytes [45], including estradiol-17β-glucuronide and estrone-3-sulfate (E1S) [46,47]. E1S is a major component of conjugated equine estrogens, the estrogen component of the predominant EPT regimens in the US (at least prior to 2002) [48]. Two [46,49] of the three [46,49,50] experimental studies reported that variant forms of *SLCO1B1* showed reduced uptake of E1S and estradiol glucuronide. Specifically, polymorphisms leading to amino acid changes within the transmembrane-spanning domains such as rs56101265 (Phe73Leu), rs56061388 (Val82Ala), rs4149056 (Val174Ala), and rs55901008 (Ile353Thr) [46,49] and those within extracellular loop 5 such as rs56387224 (Asn432Asp), rs72559748 (Asp462Gly), and rs59502379 (Gly488Ala) [49], were shown to affect the uptake kinetics. The MAF of rs4149056 (Val174Ala) was 0.14 in Europeans [49], but MAFs of the rest were less than or equal to 0.02 [49]. Rs4149056 was associated with blood E1S levels in Europeans [46]. However, rs4149056 was not in LD with rs7489119 (r^2^ = 0.01) in European populations based on HapMap data [51], and was not associated with MD change in our study using imputed genotype data. The imputation certainty and info scores for rs4149056 were 0.98 and 0.92, respectively. These observations suggest that rs4149056 is unlikely to be a causal variant of the observed association for rs7489119. We could not check LD between rs7489119 and the other functional SNPs in *SLCO1B1* because genotype data for these SNPs are not publicly available or monomorphic in Europeans [35].

More recently, using an independent data from a breast cancer case-control study nested within CTS, our group reported that EPT use modified the effect of *SLCO1B1* SNPs on breast cancer risk [29]. We found that rs4149013, a SNP located near the 5’ end of *SLCO1B1* with unknown functional significance, was associated with breast cancer risk, and this association was restricted to EPT users (odds ratio (OR) = 2.3 per minor allele) [29]. This SNP did not show an association with EPT-related MD change in the current analysis, and is not in LD with rs7489119 (r^2^ = 0.003).

Arylsulfatase C (ARSC), also known as steroid sulfatase (STS), is expressed in the liver and breast, and converts E1S into biologically active E1 [52]. Rs5933863 is located in 3’ UTR of ARSC. To our knowledge, no data exist regarding the functional significance of rs5933863 or other SNPs in ARSC.

Our current finding that *PGR* genetic variation is not associated with EPT-related MD change is consistent with the results from our own previous report based on PEPI trial data [15], although an earlier longitudinal study based on EPIC data showed that rs10895068 (+331 G/A) of the two tested *PGR* SNPs showed an association [24].

Our exploratory analysis in nulliparous women suggests that *ESR1* and *COMT* SNPs (rs2077647, and rs9605030, respectively) may be associated with EPT-associated MD changes in this subset. Although we had limited sample size for this subset analysis and these observations may be chance findings, our data are novel and warrant evaluation in larger studies of nulliparous women, who have higher MD [40,41] and higher breast cancer risk than parous women [53]. It has been hypothesized that breast tissue cells of nulliparous women may be subject to greater damage from carcinogens than the cells of parous women [42], as nulliparous breast tissue cells have not undergone parity-induced proliferation, differentiation, and involution [39].

After the publication of WHI trial results in 2002 [9], EPT use markedly dropped in the US [54]; this was immediately followed by decreases in breast cancer incidence [26,55]. It was reported that the elevated breast cancer risk in the WHI EPT group decreased rapidly after terminating the intervention, and the hazard ratio representing the risk of breast cancer associated with EPT use became approximately 1.0 within two years of cessation of exposure [56]. However, the large individual variation in MD decrease after stopping hormone therapy [18] suggests that the magnitude of risk reduction after stopping EPT may vary. Given that individual characteristics of women (for example age, BMI, parity, family history of breast cancer) are not related to the variability in MD changes [18], our findings that genetic factors may determine the amount of change in MD after quitting EPT require confirmation in future studies. Further, whether these predictors of MD decline, also predict an increase in MD when starting EPT, must be verified. The clinical management guidelines published in 2014 recommend using the lowest effective dose for the shortest duration for management of menopausal symptoms [57]. For women who consider this therapy to curtail menopausal symptoms, it would be beneficial to know whether they are at higher risk of experiencing MD changes resulting from EPT use. Such findings could help to identify a subgroup of women who should avoid EPT use.

In this study we were able to comprehensively investigate hormone metabolism pathway genes as determinants of EPT-related MD changes. A strength of our study is that one experienced investigator estimated MD in all of the mammograms using a standardized and validated method; further, both sets of mammograms from the same woman were evaluated within the same batch. A limitation of our study is that we only studied non-Hispanic white women. In addition, we did not have a comparison group who continued to use EPT or who never used EPT. Thus, it is possible that the genetic predictors of change in MD that we identified may not be specifically associated with changes following cessation of EPT, but also associated with MD reductions following aging.

## Conclusions

Data from this longitudinal study of EPT quitters suggest that genetic variation in two hormone metabolism pathway genes, *SLCO1B1* and *ARSC*, may be associated with change in MD after women quit EPT use. Larger longitudinal studies are needed to confirm our findings.

## Additional files

Additional file 1:
**Describes the characteristics of women who were initially selected for recruitment.**
Additional file 2:
**Manhattan plot showing the associations of genotyped and imputed** ***SLCO1B1*** **SNPs and MD changes.**
Additional file 3:
**Contains results of all single SNP association tests.**

## Abbreviations

ARSC: arylsulfatase C
ARTP: adaptive rank truncated product
BMI: body mass index
CEE: conjugated equine estrogens
CTS: California Teachers Study
E1: estrone
E1S: estrone sulfate
EPIC: European Prospective Investigation into Cancer and Nutrition
EPT: estrogen plus progestin therapy
LD: linkage disequilibrium
MAF: minor allele frequency
MD: mammographic density
MPA: medroxyprogesterone acetate
PEPI: Postmenopausal Estrogen/Progestin Interventions
*PGR*: progesterone receptor gene
SNP: single nucleotide polymorphism
WHI: Women’s Health Initiative
UTR: untranslated region
