# Supplementary material for: Hormone metabolism pathway genes and mammographic density change after quitting estrogen and progestin combined hormone therapy in the California Teachers Study
Source: Breast Cancer Res. 2014 Dec 11;16(6):477. doi: 10.1186/s13058-014-0477-8 (PMC4318222; doi:10.1186/s13058-014-0477-8)
Supplement: Additional file 1: — Describes the characteristics of women who were initially selected for recruitment. [file 13058_2014_477_MOESM1_ESM.docx]

Characteristics of 1,420 women who were initially selected for recruitment.

| **Characteristics at cohort enrollment** | 1,420 women selected for recruitment;  N (%) or mean ± SD |
| --- | --- |
| Age (mean ± SD) | 49.9 ± 4.2 |
| BMI (Kg/m2) | 24.5 ± 4.8 |
| White (N (%)) | 1258 (89%) |
| Nulliparous women (N (%)) | 289 (20%) |
| Menopausal status |  |
| Premenopausal | 857 (60%) |
| Perimenopausal | 190 (13%) |
| Postmenopausal | 372 (26%) |
| Ever had breast biopsy (N (%)) | 216 (15%) |
| Positive 1st degree family history of breast cancer (N (%)) | 146 (11%) |
