# Supplementary figures and images for: Hormone metabolism pathway genes and mammographic density change after quitting estrogen and progestin combined hormone therapy in the California Teachers Study
Source: Breast Cancer Res. 2014 Dec 11;16(6):477. doi: 10.1186/s13058-014-0477-8 (PMC4318222; doi:10.1186/s13058-014-0477-8)

Additional File 3. Manhattan plot for the genotyped and imputed *SLCO1B1* SNPs. Green, genotyped SNPs.

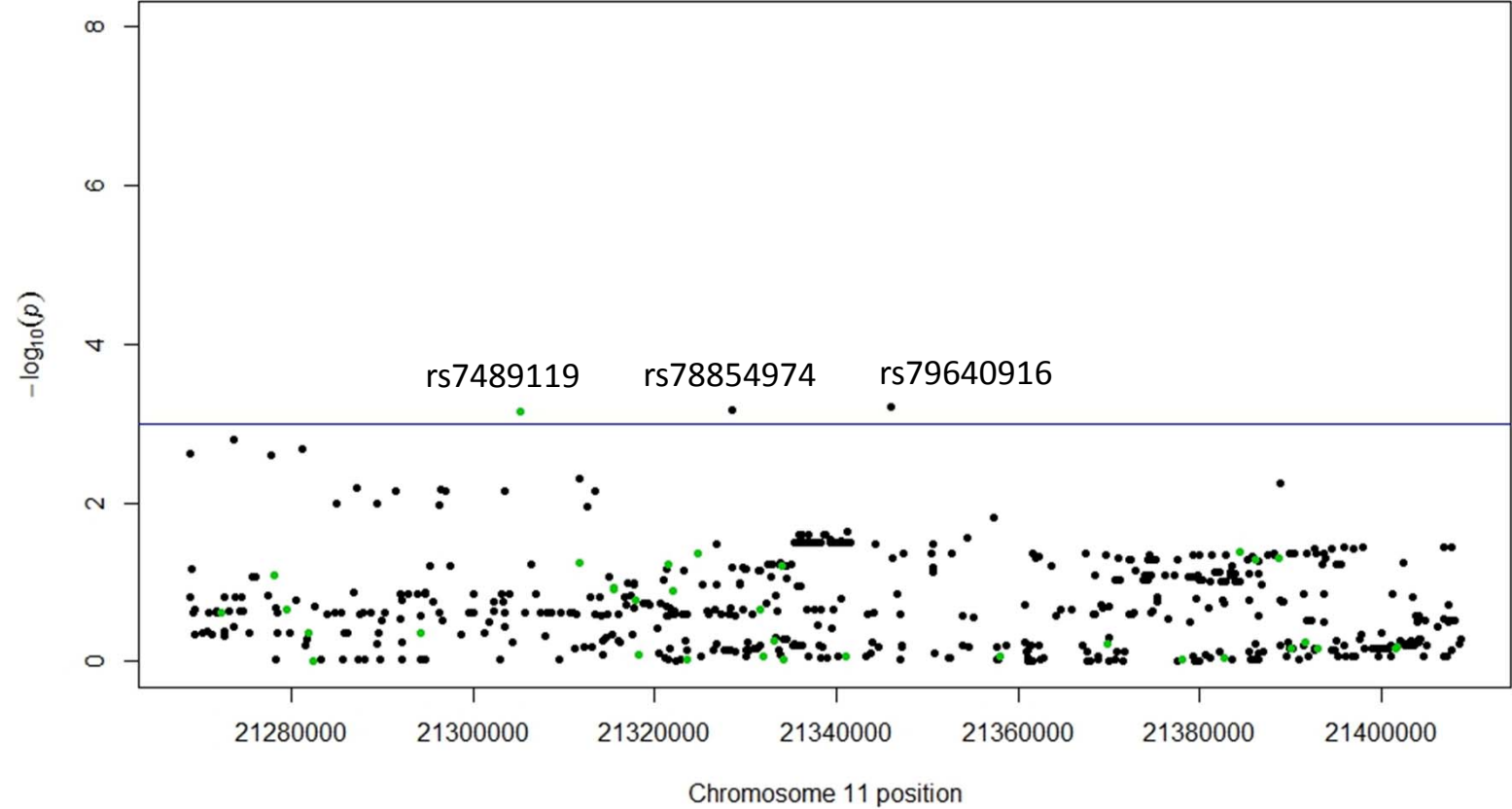

Supplement: Additional file 2: — Manhattan plot showing the associations of genotyped and imputed SLCO1B1 SNPs and MD changes. [file 13058_2014_477_MOESM2_ESM.pdf]
